# *Bacteroides vulgatus* and *Bacteroides dorei* predict immune-related adverse events in immune checkpoint blockade treatment of metastatic melanoma

**DOI:** 10.1186/s13073-021-00974-z

**Published:** 2021-10-13

**Authors:** Mykhaylo Usyk, Abhishek Pandey, Richard B. Hayes, Una Moran, Anna Pavlick, Iman Osman, Jeffrey S. Weber, Jiyoung Ahn

**Affiliations:** 1 Department of Population Health, NYU Grossman School of Medicine, New York, NY USA; 2 NYU Laura and Isaac Perlmutter Cancer Center, New York, NY USA; 3 The Ronald O. Perelman Department of Dermatology, NYU Grossman School of Medicine, New York, NY USA; 4 Department of Medicine, NYU Grossman School of Medicine, New York, NY USA

**Keywords:** Microbiome, Melanoma, Immune-related adverse events, Toxicity, Biomarkers, Survival, Prospective design

## Abstract

**Background:**

Immune checkpoint blockade (ICB) shows lasting benefits in advanced melanoma; however, not all patients respond to this treatment and many develop potentially life-threatening immune-related adverse events (irAEs). Identifying individuals who will develop irAEs is critical in order to improve the quality of care. Here, we prospectively demonstrate that the gut microbiome predicts irAEs in melanoma patients undergoing ICB.

**Methods:**

Pre-, during, and post-treatment stool samples were collected from 27 patients with advanced stage melanoma treated with IPI (anti-CTLA-4) and NIVO (anti-PD1) ICB inhibitors at NYU Langone Health. We completed 16S rRNA gene amplicon sequencing, DNA deep shotgun metagenomic, and RNA-seq metatranscriptomic sequencing. The divisive amplicon denoising algorithm (DADA2) was used to process 16S data. Taxonomy for shotgun sequencing data was assigned using MetaPhlAn2, and gene pathways were assigned using HUMAnN 2.0. Compositionally aware differential expression analysis was performed using ANCOM. The Cox-proportional hazard model was used to assess the prospective role of the gut microbiome (GMB) in irAES, with adjustment for age, sex, BMI, immune ICB treatment type, and sequencing batch.

**Results:**

Two natural GMB clusters with distinct community compositions were identified from the analysis of 16S rRNA data (*R*^2^ = 0.16, *p* < 0.001). In Cox-proportional hazard modeling, these two clusters showed a near 7-fold differential risk for developing irAEs within 1 year of initiating treatment (HR = 6.89 [95% CI: 1.33–35.58]). Using shotgun metagenomics, we further identified 37 bacterial strains differentially expressed between the risk groups, with specific dominance of *Bacteroides dorei* within the high-risk GMB cluster and *Bacteroides vulgatus* in the low-risk cluster. The high-risk cluster also appeared to have elevated expression of several functional pathways, including those associated with adenosine metabolism (all FDR < 0.05). A sub-analysis of samples (*n* = 10 participants) at baseline and 6 and 12 weeks after the start of treatment revealed that the microbiome remained stable over the course of treatment (*R*^2^ = 0.88, *p* < 0.001).

**Conclusions:**

We identified two distinct fecal bacterial community clusters which are associated differentially with irAEs in ICB-treated advanced melanoma patients.

**Supplementary Information:**

The online version contains supplementary material available at 10.1186/s13073-021-00974-z.

## Background

Immune checkpoint blockade (ICB) with therapeutics such as the anti-CTLA-4 antibody ipilimumab (IPI) and anti-PD-1 antibodies nivolumab (NIVO) and pembrolizumab have revolutionized the treatment of advanced melanoma, producing durable clinical benefit [1]. However, not all patients receiving ICB treatment benefit, and a substantial portion experience immune-related adverse events (irAEs), including colitis, diarrhea, endocrinopathies, rashes, pneumonitis, myocarditis, and hepatitis. irAEs are sometimes life-threatening, often requiring systemic immunosuppression or complete discontinuation of therapy [2]. It is urgent to identify biomarkers that predict for irAEs before the initiation of therapy, in order to implement personalized treatment to mitigate their effect.

Increasing evidence suggests that the gut microbiome (GMB) plays an important role in regulating innate and adaptive immunity [3]. In the context of ICB treatment, we [4] and others [5–7] have demonstrated that the structure of the human gut microbiome is an important pre-treatment predictor for the efficacy of ICB treatment, but the relationship of the pre-treatment microbiome with irAEs remains unclear. irAEs are generally associated with more aggressive forms of ICB treatment (i.e., combination IPI/NIVO), which in turn are associated with greater treatment efficacy [8]. Healthy donor fecal microbial transplant (FMT) into melanoma patients was reported to reverse ICB therapy-related colitis, without compromising the success of the cancer treatment [9], suggesting that GMB may be directly involved in the control of irAEs. Cross-sectional studies also have identified bacteria associated with irAEs, but the specific taxa that were present were not consistent among studies [10].

We performed a study using stool samples prospectively collected from 27 patients prior to the initiation of ICB treatment of melanoma to identify GMB risk factors for developing irAEs. We performed a thorough characterization of the GMB using three different sequencing approaches to both ensure the consistency of the results and improve the generalizability of the data by identifying the strain-level microbial differences associated with the risk of irAES. Specifically, we utilized 16S rRNA sequencing to characterize the overall GMB structure of patients. We then performed deep shotgun metagenomic sequencing in order to identify the reliable strain-level differences associated with irAEs. Furthermore, we used the shotgun data and additional confirmation with RNA-seq analysis to identify taxon independent risk markers. Finally, we used Cox-proportional hazards time-to-event analysis to relate pre-treatment GMB to irAE development. Our study suggested that elements of the GMB were associated with the development of irAES with ICB in advanced stage melanoma.

## Methods

### Study population

We studied 27 metastatic melanoma patients (stages 3–4) scheduled to receive immunotherapy at NYU Langone Health as previously described [4]. Briefly, subjects were recruited in 2016–2017 and followed for the development of outcomes until September 2018. All patients were educated about the study and provided written informed consent prior to the initiation of ICB treatment (IRB#10362) and entrance into our prospective observational study. Stool kits were provided prior to the initiation of treatment. In addition, stool collection kits were also provided at 6 and 12 weeks after treatment initiation. Survival analysis outcomes for irAEs were defined to be the time to the incidence of a grade 2+ toxicity event following initiation of ICB treatment. Grade 2 toxicity represents moderate side effects and would usually result in the suspension of ICB treatment. Examples of grade 2 toxicity include abdominal pain, mucus or blood in stool, and diarrhea frequency 4–6/day above a patient’s normal levels [11].

### Stool collection

Patients collected stool at home prior to the start of immunotherapy. Kits included a stool collection tube with 10 mL RNAlater, instructions for stool collection, and a return addressed box with pre-paid postage. Patients were instructed to mail the samples back within 1 day; upon receipt, samples were stored at − 80 °C until use. This procedure was also followed for the 6- and 12-week collection.

### 16SV4 sequencing

Stool samples underwent 16SV4 rRNA gene sequencing at the Environmental Sample Preparation and Sequencing Facility at Argonne National Laboratory, as previously described [4]. DNA was extracted using the Mo Bio PowerSoil DNA isolation kit, following the manufacturer’s protocol. The V4 region of the 16S rRNA gene was PCR amplified with the 515F/806R primer pair, which included sequencer adapter sequences used in the Illumina flow cell and sample-specific barcodes [12]. Each 25 μL PCR reaction contained 9.5 μL of Mo Bio PCR Water (Certified DNA-Free), 12.5 μL of QuantaBio’s AccuStart II PCR ToughMix (2× concentration, 1× final), 1 μL Golay barcode tagged forward primer (5 μM concentration, 200 pM final), 1 μL reverse primer (5 μM concentration, 200 pM final), and 1 μL of template DNA. The conditions for PCR were as follows: 94 °C for 3 min to denature the DNA, with 35 cycles at 94 °C for 45 s, 50 °C for 60 s, and 72 °C for 90 s, with a final extension of 10 min at 72 °C. PCR products were quantified using PicoGreen (Invitrogen) and a plate reader (Infinite 200 PRO, Tecan). Sample PCR products were then pooled in equimolar amounts, purified using AMPure XP Beads (Beckman Coulter), and then quantified using a fluorometer (Qubit, Invitrogen). Molarity was then diluted to 2 nM, denatured, and then diluted to a final concentration of 6.75 pM with a 10% PhiX spike for sequencing on the Illumina MiSeq. Amplicons were sequenced on a 151 bp × 12 bp × 151 bp MiSeq run [13].

### 16SV4 rRNA bioinformatics

Sequence reads were processed using QIIME2 [14]. Briefly, sequence reads were demultiplexed, and paired-end reads were trimmed to remove bases that fell had a PHRED quality score of 25 or lower using priseq-lite [15]. Reads were then joined using PANDAseq [16]. DADA2 [17] was then used to error correct the merged reads and to identify the amplicon sequence variants (ASVs). The DADA2 pipeline works by utilizing the error profile of the Illumina reads in order to achieve single-nucleotide resolution. This allows our 16S data to be grouped at a sequence level, which offers higher taxonomic resolution over traditional clustering into operational taxonomic units [17]. ASVs are then assigned taxonomy a bacterial taxonomy using a naïve Bayes classifier [18] pre-trained on the Greengenes 13_8 99% OTUs [19].

### Shotgun metagenomic sequencing

Stool samples underwent shotgun metagenome sequencing at the Environmental Sample Preparation and Sequencing Facility at Argonne National Laboratory. DNA was extracted as above and quantified using a fluorometer (Qubit, Invitrogen). DNA was then mechanically sheared to the desired insert size of the final library using the Covaris S-series system, and products were brought to 15 μL using Agencourt AMPure XP beads (Beckman Coulter). The Apollo 324 system (Takara Bio) was then used for end-repair, A-tailing, Illumina adaptor and barcode ligation, and size selection to generate the libraries. Libraries are run through 10–15 cycles of PCR with Kapa Biosystems Library Amplification kits, followed by further size selection with Blue Pippin Prep (Sage Science). Final library quantification is achieved using the Qubit Fluorometer (for concentration) and the Agilent 2100 Bioanalyzer (for library insert size and length). Libraries were sequenced on the Illumina HiSeq 2500 on a 2 × 101 bp paired-end run.

### Shotgun metagenomic bioinformatics

Reads were demultiplexed, and Trimmomatic [20] was used for read length filtering, trimming of Illumina adapter sequences, and trimming of low-quality read ends. Reads mapping to the human genome were identified using Bowtie2 [21] and removed. Forward and reverse reads were concatenated for input into the taxonomic and functional profiling tools, MetaPhlAn2 and HUMAnN2. MetaPhlAn2 [22] uses a set of ~ 1 million clade-specific markers (average 184 marker genes for each species) from > 7500 species to unequivocally identify and quantify specific microbial clades at the species level or higher. Reads belonging to either *Bacteroides dorei* or *Bacteroides vulgatus* were extracted from raw fastqs using Bowtie2 [21]. Reads were then assembled into contigs using metaSPADEs with default settings [23]. Final genome assembly and annotation were performed using PATRIC [24].

### Statistical analysis

All data analysis was performed using R [25]. Beta diversity was calculated employing the package using Jensen-Shannon divergence (JSD) distances [26]. The Ward.D2 clustering algorithm was used to identify sample enterotypes using the JSD distances [27, 28]. Cox-proportional hazards models were used to determine whether microbiome enterotypes were associated with the development of a grade 2+ toxicity event within 1 year of the start of treatment. PERMANOVA within the *vegan* [29] package was used to determine the significance of enterotype clustering, and kernel-based regression from the *MiRKAT* [30] package was used to adjust for covariates. The adjustment was made for age, sex, BMI, cancer stage, sequencing batch in survival, and beta diversity analysis. The Wilcoxon test was used to identify differentially abundant genera within the amplicon data. ANCOM [31] was used to identify species significantly differentially abundant between the identified enterotypes within shotgun data.

## Results

### GMB risk clusters

**Fig. 1:**
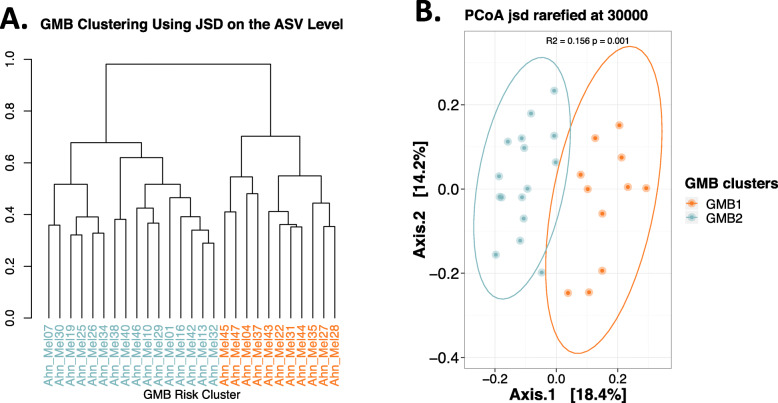
Gut microbiome clustering at the ASV level reveals two distinct communities. **A** Hierarchical clustering of samples based on the JSD distance matrix made using the relative abundance of ASVs reveals two primary clades designated as high risk (GMB1, shown in orange) and low risk (GMB2, shown in blue). **B** PCoA visualization of the JSD matrix. PERMANOVA analysis results (top of PCoA figure) added to quantify the amount of variance explained by ASV level clustering show a significant association between the GMB1 and GMB2 groups; *R*^2^ = 0.16, *p* < 0.001

Patients with metastatic melanoma (*n* = 27), scheduled to receive immunotherapy at NYU Langone Health from September 2016 to November 2017, provided stool samples at three time points (pretreatment [baseline], during [6 weeks], and after [12 weeks] treatment). The majority of the patients were male (78%) and Caucasian (96%), and 56% of the patients were receiving IPI+NIVO treatment, with the remainder receiving NIVO alone. We completed fecal 16S V4 rRNA gene amplicon and recovered an average of 71,152 reads ± 13,485, per sample, across 2209 amplicon sequence variants (ASVs) that represented 217 unique genera and 123 species. Based on unsupervised hierarchical clustering at the amplicon sequence variant (ASV) level, two distinct clusters (one designated as “GMB1” and shown in orange and the other designated as “GMB2” and colored as blue) were identified (Fig. 1A). These clusters represented significant differences in the overall GMB structure, based on unadjusted PERMANOVA analysis (*R*^2^ = 0.16 *p* < 0.001; Fig. 1B) and with kernel-based covariate adjustment for subject age, sex, BMI, and treatment type (*p* < 0.001).

**Fig. 2:**
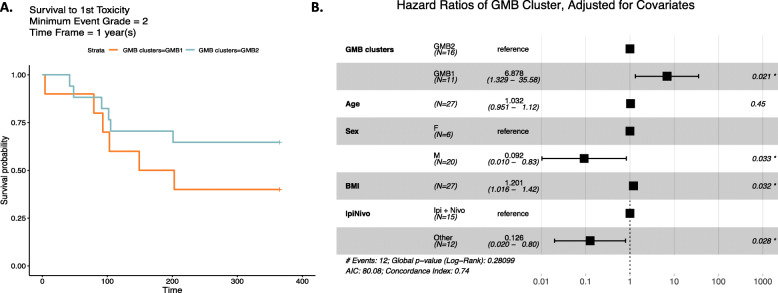
Toxicity-free survival using GMB clusters. **A** Kaplan-Meier curve is shown for the survival to 1st grade 2 (or higher) toxicity event. The GMB2 GMB cluster is shown in blue, and the GMB1 GMB cluster is shown in orange. **B** A forest plot showing the results of the proportional hazards regression model analysis with the GMB clusters serving as the predictor with adjustment for the subject age, sex, BMI, and ICB treatment. The results indicate a significantly higher risk of suffering a grade 2 toxicity even based on belonging to the GMB1 cluster; HR = 6.88 [95% CI: 1.33–35.58], *p* = 0.021

We examined whether these naturally occurring GMB clusters were associated with immune-related adverse events (irAEs). In Kaplan-Meir analysis, we showed that irAE grade 2+ occurred more frequently for study participants in the GMB1 (orange, *n* = 11) versus the GMB2 (blue, *n* = 16) group (Fig. 2A, *p* = 0.021). In Cox-proportional hazard analysis, we showed that the adjusted risk of an adverse event (irAE grade 2+) was nearly 7-fold greater for the GMB1 than the GMB2 group (hazard ratio (HR) = 6.88 [95% CI: 1.33–35.58] *p* = 0.021) (Fig. 2B, data adjusted for age, sex, BMI, and immunotherapy type). Males appeared to be less likely to develop irAEs compared to women, HR = 0.092 [95% CI: 0.010–0.83] *p* = 0.033; BMI on a continuous scale was positively associated with irAEs, HR = 1.20 [95% CI:1.02–1.42] *p* = 0.032; and, consistent with previous studies [32], mono-antibody treatment had an overall lower risk of irAEs, HR = 0.13 [95% CI: 0.020–0.80] *p* = 0.028.

**Fig. 3:**
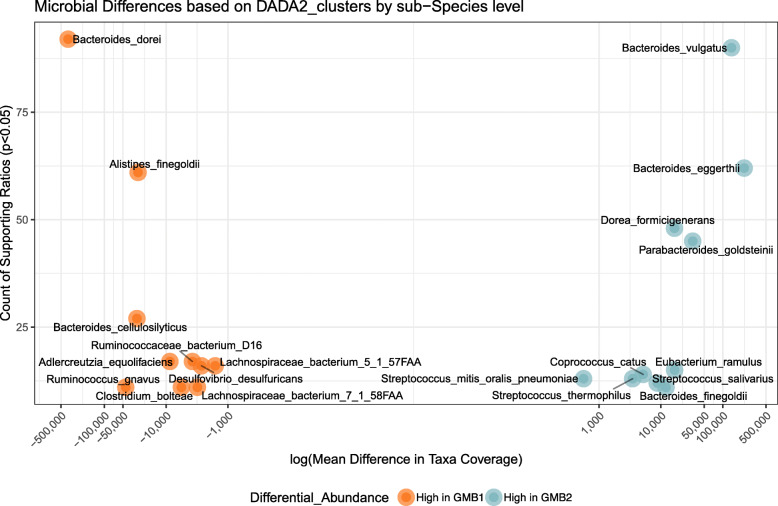
Bacterial species associated with GMB risk clusters. Scatter plot shows the bacteria that are significantly increased in either the HR (orange) or LR (blue) clusters. The *y*-axis shows the ANCOM W-stat, which indicates the number of microbial reference frames that were significantly differentially abundant between the GMB clusters (FDR < 0.05). The *x*-axis shows the mean difference in shotgun reads between the two risk clusters. ANCOM analysis was performed with adjustment for age, sex, BMI, and ICB treatment

In order to identify what was driving the composition of these distinct naturally occurring GMB risk clusters, we analyzed the top ASVs identified using DADA2 analysis (Additional file 1, Fig S1). There appeared to be two unclassified sequence clusters belonging to the Genus *Bacteroides* which uniquely dominated each of the identified GMB clusters; that is, the GMB2 and GMB1 clusters were both dominated by *Bacteroides* but by different strains of this genus (Additional file 1, Fig S1). In order to resolve the dominant taxa at the strain level, we performed compositionally aware differential abundance analysis using ANCOM, employing our shotgun metagenomic sequencing data. The data suggested that the two distinct clusters associated with irAEs were largely attributed to two distinct strains of the *Bacteroides* genus (Fig. 3); *Bacteroides vulgatus* was dominant in the GMB2 group and *Bacteroides dorei* in the GMB1 group (effects adjusted for patient age, sex, BMI, and ICB treatment type, FDR < 0.001). Based on the shotgun metagenomic sequencing analysis, we determined that within the GMB1 cluster, *B. dorei* accounted for an average of 7.5% ± 9.1% shotgun reads vs. 0.50% ± 0.57% in the GMB2 group. In contrast, *B. vulgatus* was more dominant in the GMB2 group with an average read count of 3.1% ± 2.2% compared to 0.64% ± 1.1% in the GMB1 group. The full ANCOM analysis revealed an additional 37 strains identified to be differentially abundant between the two GMB risk clusters (W-stat > 10, Additional file 1, Table S1). Figure 3 shows the top 20 strains ranked, based on the number of validating microbial ratios. To further test whether the two distinct species act as risk factors for irAE, we created an additional Cox-proportional hazard model that considered the log ratio of the two bacteria as the core predictor, with adjustment for the patient age, sex, BMI, and ICB treatment. The results validated the cluster results with the tested bacterial ratio presenting an adjusted hazard ratio of 1.20 [1.004–1.40] *p* = 0.046.

**Fig. 4:**
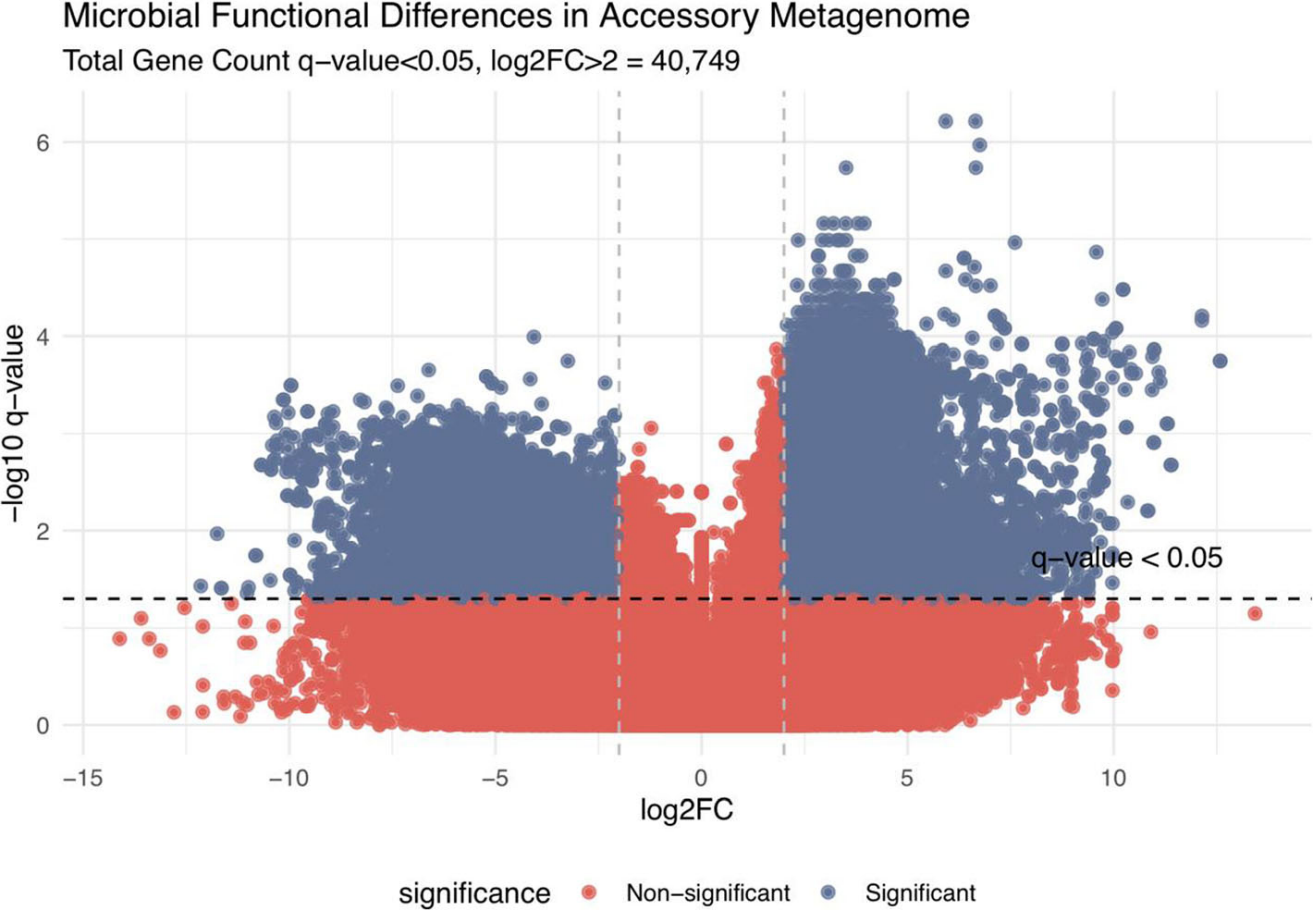
Volcano plot showing differentially abundant genes between the GMB risk clusters. Volcano plot shows the top 25% of most variant genes in terms of RPK. Genes were stratified by the microbial strain of origin when a confident identification could be made using HUMAnN 2.0. Genes were considered to be significantly differentially abundant when the FDR < 0.05 and the log2FC > 2.0. The *x*-axis in the plot indicates which GMB cluster the genes were elevated (left for GMB2 and right for GMB1). The *y*-axis shows the log of the significance level. The results show that 22,985/40,749 were significantly elevated within the GMB1 cluster and only 17,764/40,749 within the GMB2 cluster

**Fig. 5:**
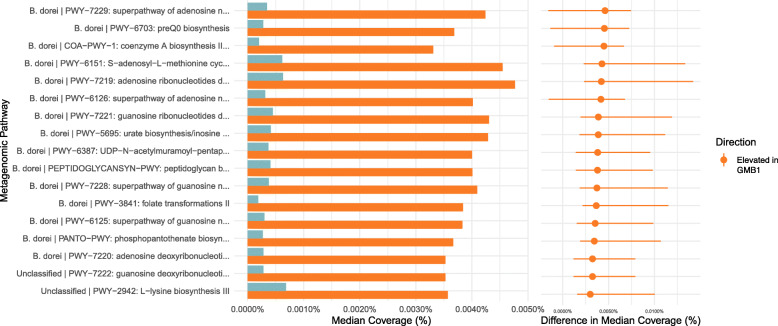
Differentially abundant MetaCyc pathways. HUMAnN2-derived genes were collapsed into functional pathways using MinPath and analyzed for differential expression. Of the 2251 analyzed pathways, 17 were significant (FDR < 0.05) and are shown in the figure. Bar plot shows the median RPK values found within each group with a difference in the median coverage presented as a dot plot with associated 95% confidence intervals

In order to identify taxon-independent markers associated with GMB risk clusters, we performed differential abundance analysis of the identified genes. In order to remove core genes from the analysis, gene variance was computed for all remaining genes, and the top 25% of the genes were used for final analysis. Figure 4 shows a volcano plot that displays a total of 40,749 genes differentially abundant between the two risk clusters (median log2FC > 2, *q*-value < 0.05, Additional file 1, Fig S2). To validate the metagenomic data, we performed a sub-analysis using the RNA-seq data by comparing the normalized RPK values of all of the identified genes in a random subset of 18 samples. Additional file 1, Fig S3 shows the paired results from the RNA- and DNA-based assays and suggests a good level of concordance with an overall median correlation of 0.753 [95% CI 0.426–0.906] for the 40,749 genes that were differentially expressed between the GMB risk clusters. In order to identify the consequence of this differential expression on a more biologically relevant scale, the genes were organized into MetaCyc pathways. Of the 2251 analyzed pathways, 17 showed significant differential expression across the GMB clusters (FDR < 0.05). Figure 5 presents these pathways, all of which were elevated in the GMB1 cluster (designated in orange). Five of the 17 pathways associated with the GMB1 cluster were related to adenosine metabolism: PWY-7219, adenosine ribonucleotides de novo biosynthesis; PWY-7229, superpathway of adenosine nucleotides de novo biosynthesis I; PWY-6126, superpathway of adenosine nucleotides de novo biosynthesis II; and PWY-7220, adenosine deoxyribonucleotides de novo biosynthesis II.

### Sub-analysis: GMB stability

In order to determine whether the GMB clusters remained stable over the course of the ICB treatment, we profiled the GMB of 10 samples at baseline and 6 and 12 weeks using 16S rRNA sequencing, as described for the main analysis. The baseline gut microbiome was stable through the course of immunotherapy treatment over 12 weeks (Additional file 1, Table S1, JSD *R*^2^ = 0.88 *p* < 0.001 and Jaccard *R*^2^ = 0.67 *p* < 0.001). Similarly, the alpha diversity analysis showed no significant differences across the three time points by either Chao1 (Kruskal-Wallis *p* = 0.59) or Shannon measures (Kruskal-Wallis *p* = 0.63). Beta diversity analysis across visits showed no significant differences between visits by either distance measure, JSD *R*^2^ = 0.005 *p* = 1.00 and Jaccard *R*^2^ = 0.032 *p* = 1.00 (Additional file 1, Fig S4A and SB).

## Discussion

We identified two natural clusters of the gut microbiome, by using unsupervised cluster analysis, in melanoma patients receiving treatment with immune checkpoint blockade (ICB). One cluster was represented by a high abundance of *Bacteroides dorei*, and the other was represented by a high abundance of *Bacteroides vulgatus*. On follow-up, patients whose gut microbiome at baseline was characterized by a high abundance of *Bacteroides dorei* were at high risk (GMB1) for immune-related adverse events (irAE 2+), compared to patients characterized by high abundance of *Bacteroides vulgatus* (GMB2, low-risk group). The microbial profiles of these distinct clusters were related to differential expression of several microbial gene functional pathways; in particular, enzyme capacity for adenosine metabolism was over-represented in the GMB1 cluster. We further showed that these baseline pretreatment gut microbiome species remained stable over follow-up, even with immunotherapy.

Our results are in line with several studies involving both human subjects [33–35] and mouse models [36, 37], where species within the genus *Bacteroides* are identified in association with the incidence of irAEs. *Bacteroides* species may act to maintain a state of continuous systemic inflammation [38] which may be part of the causal pathway for irAEs. This notion is further supported with studies showing that the dominance of *Bacteroides dorei* (dominant strain within the high-risk GMB1 cluster) is the dominant GMB constituent directly preceding an autoimmune response in a prospective cohort of children at risk of type 1 diabetes [39]. It may be the case that the dominance of this particular strain of *Bacteroides* presents a non-specific elevation of risks towards various types of immune reactions. ICB treatment may therefore act to initiate this patient-specific microbial state.

Not all species of *Bacteroides*, however, are associated with increased risk for irAE development. Here, we show, for the first time, that *Bacteroides vulgatus* is associated with a lowered risk of irAE, despite being closely related phylogenetically to *Bacteroides dorei* [40]. Recently, Huang et al demonstrated that *B. vulgatus* is associated with improved response to ICT treatment in a mouse model [41]. Strikingly, the researchers were able to demonstrate in this study that the response effect could be transferred to mice with a poor response via a fecal microbiome transplant. Furthermore, *B. vulgatus* was also increased in non-small cell lung cancer patients that did not experience dermatological irAE, following PD1-based ICB treatment [42].

Other studies of GMB and ICB treatment outcomes have also identified the involvement of the genus *Bacteroides*. For example, *Bacteroides fragilis* appears to be commonly associated with decreased systemic inflammation via recruitment of T-reg cells [43] and elevation of the anti-inflammatory cytokine IL-10 [44]. These studies are of further interest because the research groups found that the *Bacteroides*-associated decrease in risk with irAEs was independent of ICB treatment efficacy [45]. This is critical because ICB outcome and irAE development have been shown to have a positive correlation in some histologies [46]. It therefore seems promising that GMB-targeted therapies may allow for a positive clinical outcome while also mitigating irAE. In fact, one study has already demonstrated that a fecal microbiome transplant from healthy donors can reverse the symptoms of treatment-induced colitis without impacting the efficacy of treatment [9].

Some studies identifying species of *Bacteroides* in immunoactive roles suggest the potential involvement of NOD2. This receptor plays a critical role in immune system function by acting as a microbial sensor. It is triggered by muramyl-dipeptide, a component of the bacterial cell wall found in all bacteria [47]. In the context of the NOD2 sensing pathway, *B. vulgatus* was shown to activate an immune response by translocation to the small intestine, in a mouse model that lacked functionality in NOD2 [48]. This is relevant because it indicates that the activity of species like B. vulgatus may be dependent on host genetics. *B. dorei* has also been suggested to have immune activity via NOD2 interaction in the context of autoimmune diabetes [49]. These findings are of importance because they indicate that the activity of bacteria may be dependent on specific host genetics.

Although our study is in line with others that report the activity of *Bacteroides* with regard to ICB outcomes in melanoma [33–35], other groups have proposed other markers for treatment response, such as *Ruminococcaceae* and *Faecalibacterium* [5]. A possible explanation of this discrepancy is a geographic variance of the GMB which may limit generalizability across diverse geographic regions [50]. Geography also accounts for differential distribution of enterotypes [51] and may act as an effect modifier for certain taxa when considering biomarkers of irAE. In the context of the global spectrum of human enterotypes, our data represents samples that are dominated by species of the genus *Bacteroides*. It has been reported that Western populations generally tend to have enterotypes dominated by *Bacteroides*, *Prevotella*, or *Firmicutes*. When interpreting our data, it is therefore critical to keep in mind the focus on this specific class of individuals where *Bacteroides* species are the dominant taxa.

In contrast with taxonomic biomarkers, functional microbiome profiles have been shown to be fairly conserved between populations, as shown in the Human Microbiome Project [52]. For this reason, we used shotgun metagenomics to demonstrate that the naturally occurring GMB clusters, which were associated with elevated risk for developing irAE, were differentiated by the statistically significant elevation of 2905 genes in the GMB1 group vs. the 109 genes that were elevated in the GMB2 group. Pathway analysis also identified significant differential expression between the GMB clusters. Specifically, we identified the upregulation of four pathways associated with adenosine metabolism within the GMB1 cluster. Adenosine signaling is known to be associated with the suppression of tumor immunity and has been targeted as part of ongoing clinical trials [53]. Furthermore, adenosine is currently being targeted as a biomarker for toxicity in PD-1 and CTLA4 ICB treatment [54]. In this context, host adenosine is known to act as a potent inhibitor of the immune response [54]. Given the association with the GMB1 cluster in our cohort, it may be that the continual exposure to *B. dorei* acts to increase immunotoxicity via regulation of the adenosine signaling pathway, resulting in increased irAEs, as we observed. It is important to note that our finding is based on an analysis of the microbial DNA and that further mass-spectrometry-based untargeted metabolomic analysis could serve to solidify our observations on the role of adenosine as a risk factor in irAEs. Additionally, work is warranted to explore this association in the context of a larger melanoma patient population as the GMB may be directly affecting this pertinent host signaling pathway.

Our data provide evidence that GMB composition prior to the initiation of ICB treatment is associated with the likelihood of developing irAEs. Our sub-analysis that considered GMB samples at 6 and 12 weeks after the initiation of treatment indicated that the GMB appears to be unaffected by ICB treatment. These data indicate that baseline GMB presents a continuous exposure that tends to be maintained throughout ICB treatment in metastatic melanoma. Given the evidence in a small trial that showed the benefit of a fecal microbiome transplant (FMT) [9] in reversing irAEs in patients undergoing ICB, it would be relevant to identify whether FMT patients exhibit a similar GMB trajectory. This would help to determine whether the stability of the GMB as an exposure is critical for lowering risk, or if acute inoculation can achieve the same beneficial effect.

A strength of our study is the use of multiple platforms to define components associated with the development of irAE within advanced stage melanoma patients receiving either single PD-1 antibody or combination ICB treatment. We used deeply sequenced 16S rRNA amplicon, shotgun metagenomic, and RNA-seq data (the latter to confirm results from the functional analysis). Both amplicon and shotgun data identified differences in risk due to the presence of closely related species of *Bacteroides* that are significantly associated with the development of irAEs following ICB treatment. Another strength of our study is the follow-up of patients for a year after ICB treatment initiation. This allowed us to use time-to-event analysis to prospectively demonstrate that components of the GMB are risk factors for irAE. The use of multiple samples across different time points also allowed us to demonstrate that GMB tends to remain stable throughout the course of treatment. This finding may be of critical importance if GMB manipulation is to be used to prophylactically prevent irAEs.

Our study is, however, limited by the small sample size, limiting our statistical power for discovering microbial taxa related to the small effect size. In addition, given this is an observational study, we recognize that there is a potential for residual confounding related to other unmeasured clinical variables [55]. Further microbiome studies based on a large clinical trial are warranted.

## Conclusions

In conclusion, we present evidence using three independent sequencing approaches showing that the pre-treatment GMB predicts which patients with metastatic melanoma will develop grade 2+ immune-related adverse events in contrast to those who will not. Multiple sampling across the course of treatment allowed us to demonstrate that GMB composition tends to remain stable throughout treatment, making it a promising target for prophylactic intervention, before and during the course of treatment.

## Supplementary Information

**Additional file 1: **Supplemental figures and tables are available within the “Additional file 1.docx” file which contains 16S rRNA OTU ASV variant analysis (**Fig S1**), differential clustering based on RNAseq expression levels (**Fig S2**), correlation between metagenome and metatranscriptome data (**Fig S3**), temporal stability of GMB PCoAs (**Fig S4**) and temporal stability PERMANOVA results (**Table S1**).

## Data Availability

All sequence data is available for direct download within the Sequence Read Archive (SRA) database with the BioProject accession number PRJNA541981 (https://www.ncbi.nlm.nih.gov/sra/?term=PRJNA541981) [56].
