# Supplementary material for: Bacteroides vulgatus and Bacteroides dorei predict immune-related adverse events in immune checkpoint blockade treatment of metastatic melanoma
Source: Genome Med. 2021 Oct 13;13:160. doi: 10.1186/s13073-021-00974-z (PMC8513370; doi:10.1186/s13073-021-00974-z)
Supplement: Supplementary file 1 — Additional file 1: Supplemental figures and tables are available within the “Additional file 1.docx” file which contains 16S rRNA OTU ASV variant analysis (Fig S1), differential clustering based on RNAseq expression levels (Fig S2), correlation between metagenome and metatranscriptome data (Fig S3), temporal stability of GMB PCoAs (Fig S4) and temporal stability PERMANOVA results (Table S1). [file 13073_2021_974_MOESM1_ESM.docx]

**Fig S1. Top 20 ASVs with FDR adjusted Differential Abundance Analysis**

Top 20 DADA2 ASVs were analyzed for differential abundance between the identified HR and LR groups. Two distinct unclassified species of the Bacteroides genus appear to be differentially abundant; one in the LR group and one in the HR group (FDR<0.001).

**Fig S2. Heatmap Showing Differentially Expressed Genes Between GMB Risk Clusters**

All genes with significant differential expression between the HR and LR GMB cluster (log2FC>2, FDR<0.05) are plotted as a heatmap. Hierarchical clustering reveals a strong concordance between taxonomic sample grouping and one based on functional analysis, with the exception of two samples.

**Fig S3. Correlation between Shotgun Metagenomic and RNA-seq gene RPKs**

Paired correlation between all genes found to be differentially abundant between the GMB risk cluster is presented using a scatter plot. Regression line is fitted using the least means squares method. Overall the metagenomic data appears to be strongly correlated with the RNA-seq results with a median correlation being 0.753 [95% CI: 0.426-0.906].

**Fig S4. Beta Diversity Measures Across Three Visits**

1. Shows the PCoA plot constructed using 10 longitudinal sampling at Baseline, Week 6 and Week 12 using JSD distances. PERMANOVA analysis indicates that there is no significant association between sampled visit and the observed microbial community, R^2^ = 0.005 p=1.00.
2. An alternative measure to JSD was used to further validate lack of visit effect. Jaccard distance considers presence/absence species data and would better pick up fluctuations in rare species. Similarly to JSD analysis there was no observable effect of visit on the observed microbial community based on the PERMANOVA analysis, R^2^ = 0.032 p=1.00.

**Table S1. Beta Diversity of Longitudinal Samples**

| **Distance Measure** | **Longitudinal Comparison** | **R2** | **p-val** |
| --- | --- | --- | --- |
| JSD | All Samples | 0.88 | <0.001 |
|  | Baseline - Week6 | 0.92 | <0.001 |
|  | Week6 - Week12 | 0.92 | <0.001 |
|  | Baseline - Week12 | 0.89 | <0.001 |
| Jaccard | All Samples | 0.67 | <0.001 |
|  | Baseline - Week6 | 0.78 | <0.001 |
|  | Week6 - Week12 | 0.75 | <0.001 |
|  | Baseline - Week12 | 0.72 | <0.001 |
